# Identification of active *Plasmodium falciparum* calpain to establish screening system for *Pf*-calpain-based drug development

**DOI:** 10.1186/1475-2875-12-47

**Published:** 2013-02-04

**Authors:** Byoung Yul Soh, Hyun-Ok Song, Yoonji Lee, Junghyun Lee, Kusuma Kaewintajuk, Binna Lee, Yun-Young Choi, Jeong Hoon Cho, Sun Choi, Hyun Park

**Affiliations:** 1 Laboratory of Cell & Molecular Biology, Department of Life Science, Seonam University, 590-711, Namwon, Jeonbuk, Republic of Korea; 2 Zoonosis Research Center, Department of Infection Biology, Wonkwang University School of medicine, 570-749, Iksan, Jeonbuk, Republic of Korea; 3 National Leading Research Laboratory (NLRL) of Molecular Modeling & Drug Design, College of Pharmacy, Division of Life and Pharmaceutical Sciences, and National Core Research Center for Cell Signaling and Drug Discovery Research, Ewha Womans University, 120-750, Seoul, Republic of Korea; 4 Division of Biology Education, College of Education, Chosun University, 501-759, Gwangju, Republic of Korea

**Keywords:** Calpain, Malaria, *Plasmodium falciparum*, Monomer, Cysteine protease, Protease inhibitor, Anti-malarial drug

## Abstract

**Background:**

With the increasing resistance of malaria parasites to available drugs, there is an urgent demand to develop new anti-malarial drugs. Calpain inhibitor, ALLN, is proposed to inhibit parasite proliferation by suppressing haemoglobin degradation. This provides *Plasmodium* calpain as a potential target for drug development. *Pf*-calpain, a cysteine protease of *Plasmodium falciparum*, belongs to calpain-7 family, which is an atypical calpain not harboring Ca^2+^-binding regulatory motifs. In this present study, in order to establish the screening system for *Pf*-calpain specific inhibitors, the active form of *Pf*-calpain was first identified.

**Methods:**

Recombinant *Pf*-calpain including catalytic subdomain IIa (r*Pf*cal-IIa) was heterologously expressed and purified. Enzymatic activity was determined by both fluorogenic substrate assay and gelatin zymography. Molecular homology modeling was carried out to address the activation mode of *Pf*-calpain in the aspect of structural moiety.

**Results:**

Based on the measurement of enzymatic activity and protease inhibitor assay, it was found that the active form of *Pf*-calpain only contains the catalytic subdomain IIa, suggesting that *Pf*-calpain may function as a monomeric form. The sequence prediction indicates that the catalytic subdomain IIa contains all amino acid residues necessary for catalytic triad (Cys-His-Asn) formation. Molecular modeling suggests that the *Pf*-calpain subdomain IIa makes an active site, holding the catalytic triad residues in their appropriate orientation for catalysis. The mutation analysis further supports that those amino acid residues are functional and have enzymatic activity.

**Conclusion:**

The identified active form of *Pf*-calpain could be utilized to establish high-throughput screening system for *Pf*-calpain inhibitors. Due to its unique monomeric structural property, *Pf*-calpain could be served as a novel anti-malarial drug target, which has a high specificity for malaria parasite. In addition, the monomeric form of enzyme may contribute to relatively simple synthesis of selective inhibitors.

## Background

Malaria is widespread in tropical and subtropical regions, including parts of the Americas, Asia and Africa. An estimated three billion people were at the risk of malaria and half to one million deaths were reported in 2010 [1]. Most deaths by malaria are caused by *Plasmodium falciparum*, one of the five species of human infectious malaria parasites, and malaria is related to the distribution of *Anopheles* mosquitoes [2]. Unfortunately, the increasing resistance of malaria parasites to available drugs has being reported [3,4]. Therefore, there is a need to develop new anti-malarial drugs.

*Plasmodium falciparum* invades erythrocytes and consumes the available haemoglobin as a means to obtain nutrients during growth and maturation [5]. Many *Plasmodium* proteases appear to play key roles during the life cycle of malaria, including: 1) invasion of an erythrocyte, 2) degradation of haemoglobin, and 3) rupture of erythrocytes. The degradation of haemoglobin occurs in the acidic food vacuole (FV) formed by the parasite in an erythrocyte, and up to 80% of haemoglobin is consumed by malarial parasites [2,6]. In *P. falciparum*, three different classes of proteases are mainly responsible for the haemoglobin degradation; they include aspartic proteases (plasmepsin I, II, IV and HAP), cysteine proteases (falcipain-1, -2 and −3) and the metalloprotease (falcilysin) [6-9]. Several exopeptidases such as dipeptidyl aminopeptidase 1 (DPAP1) and three metallo-aminopeptidases (A-M1, APP and LAP) have also essential roles in haemoglobin degradation [10-12]. Plasmepsin is known to be synthesized in an inactive precursor form (membrane-bound proplasmepsin), and to be processed to mature form by mature plasmepsin and falcipain, a cysteine protease [13,14]. Thus, an aspartic protease inhibitor, Pepstatin A has been known to block the processing of plasmepsin [5,6,14,15]. Since *Plasmodium* plasmepsin and falcipain are involved in haemoglobin degradation, which is necessary for parasite proliferation in the host, they have been targeted for development of anti-malarial drugs for decades [5,16-19]. However, plasmepsin activation does not seem to be completely blocked by inhibitors of aspartic proteases and/or cysteine proteases [5,20]. Recently, ALLN, a calpain inhibitor has been proposed to have the inhibitory effect of plasmepsin and falcipain [14,15]. Although its antimalarial activity is likely due primarily to the inhibition of falcipain, it still opens the possibility that calpain could be the one of the mediators for haemoglobin degradation and, thereby, a potential anti-malarial drug target.

Calpain is a cytoplasmic Ca^2+^-dependent, non-lysosomal cysteine protease that is ubiquitously expressed in mammals and many other organisms [13]. The *P. falciparum* genome encodes a single calpain homologue, although no biochemical data are available and it is not clear whether the calpain is expressed or catalytically active in any parasitic stage [8]. The *P. falciparum* calpain (*Pf*-calpain) gene differs significantly from those found in vertebrates to date [21]. A putative calpain (MAL13P1.310) in *P. falciparum* has high sequence similarity to *Caenorhabditis elegans* calpain-7 [22-24]. They belong to a monophyletic group of calpain-7, which might have contributed to an alternative Ca^2+^-independent calpain activity [22]. *Pf*-calpain consists of a central catalytic domain II (subdomain IIa and IIb) and a C-terminal catalytic domain III. This domain composition is a distinct type that is not common to any other types of calpain classes [24]. *Pf*-calpain was believed to be an essential mediator of merozoite invasion, based on the observation that a calpain inhibitor blocked invasion [25]. As the parasite progresses from trophozoite to schizont stage, there is a 30-fold increase in the level of calpain transcription [22].

Based on reports, *Pf*-calpain seems to play a role in haemoglobin degradation along with plasmepsin and falcipain [14,15,26]. However, no direct evidence was proposed that *Pf*-calpain participates in haemoglobin degradation and ALLN indeed acts against this enzyme. Thus, in this study, the active form of *Pf*-calpain was investigated in the first place to check its enzymatic activity and inhibition by ALLN. This active *Pf*-calpain was further utilized to establish the high-throughput screening system for *Pf*-calpain inhibitors. Results suggest that *Pf*-calpain is active only with catalytic subdomain IIa, resulting in a monomeric form of enzyme. In addition, the enzymatic activity of *Pf*-calapin was efficiently inhibited by ALLN treatment. The monomeric structure of *Pf*-calpain is considerably distinct from mammalian typical calpains and, thereby, *Pf*-calpain could serve as a target to develop parasite specific anti-malarial drugs. In addition, the monomeric structure might accelerate drug development by simplifying the synthesis steps of *Pf*-calpain selective inhibitors.

## Methods

### Construction of recombinant calpains and point-mutated calpains

The calpain genes were amplified using full length DNA isolated from *P. falciparum* strain FCR-3. The calpain genes for recombinant proteins were amplified by PCR using the following primers: r*Pf*cal-IIa; forward (5′-*CGG GAT CCC* GGA ATG GGT AAA AGC AAA GAA CGT AAA GGT-3′) and reverse (5′-*CCG CTC GAG CGG* CTT TGT GTC CTC TAC AAA TTC AAC ACT GTT-3′), r*Pf*cal-IIb; forward (5′-*CGG GAT CCC* AAC GGG TCA GTG GAT AAT TAT AGT GAT TTG-3′) and reverse (5′-*CCG CTC GAG CGG* ATC CAC ATT ATT CAC ATT ATC CAC ATT ATC CAC-3′), r*Pf*cal-IIab; forward (5′-*CGG GAT CCC* GGA ATG GGT AAA AGC AAA GAA CGT AAA GGT-3′) and reverse (5′-*CCG CTC GAG CGG* ATC CAC ATT ATT CAC ATT ATC CAC ATT ATC CAC-3′). The forward primers contained *Bam*HI site (in italics) and reverse primers contained XhoI site (in italics) at their respective 5′-end. The PCR product was cloned into the pGEM-T easy vector and digested with BamHI and XhoI (New England BioLabs Inc., USA). The calpain gene was ligated into pET21b(+) vector (Novagen, USA) following its digestion with BamHI and XhoI. The resultant pET21b: r*Pf*cal-IIa plasmid was further used as a parent construct for alanine-substitution mutants. Alanine-substitution mutants at appropriate positions of r*Pf*cal-IIa were constructed using overlapping PCR. To generate recombinant baculoviruses harboring calpain genes, wild type and mutant calpain genes were amplified by PCR using pET21b::r*Pf*cal-IIa and pET21b::r*Pf*cal-IIa(HN) plasmid as parent constructs. The following primers were used to construct: forward (5′-AT CAC CAT AC*G GAT CC*C GAA GGA ATG-3′) and reverse (5′-G GGT ACC CCG AT*C TGC AG*T ATT CAC ATT-3′). All primers contained BamHI or PstI restriction sites (shown as italic) for ligation to the pFastBac HTA-M vector, kindly provided by Dr. JM Yang in Sogang University, Korea.

### Expression and purification of recombinant calpain proteins

#### Bacterial system

The recombinant constructs was transformed into *Escherichia coli* BL21 (DE3) cells. Induction was performed with 1 mM isopropyl-β-D-thiogalactopyranoside (IPTG) for four hours. Cells were harvested by centrifugation and resuspended in 6 M Gu-HCl, 0.1 M sodium phosphate buffer, 0.01 M Tris-Cl, pH 8.0 for 60 min. The cell lysate was centrifuged and the supernatant was incubated with the 50% Ni-NTA slurry for 60 min at room temperature. The protein-bound resin was loaded onto a column and washed twice with 4 ml of 8 M Urea, 0.1 M sodium phosphate buffer, 0.01 M Tris-Cl, pH 6.3. The bound proteins were eluted with 8 M Urea, 0.1 M sodium phosphate buffer, 0.01 M Tris-Cl, pH 5.9 and continuously with 8 M Urea, 0.1 M sodium phosphate buffer, 0.01 M Tris-Cl, pH 4.5. The eluted proteins were quantified using the Bradford protein assay (Bio-Rad, USA) and analysed by SDS-PAGE and Western blot. r*Pf*-calpain proteins were processed for refolding by two-step dialysis in 0.01 M Tri-HCl, pH 7.5.

#### Insect cell system

The recombinant pFastBac HTA-M plasmids were transfected into *E. coli* DH10Bac cells (Invitrogen, USA) to induce the transposition of insert into baculoviral shuttle vector. The resultant recombinant baculoviruses were transfected to Sf9 cells (Invitrogen, USA) treated with VivaMagic^TM^ Transfection Reagent (Vivagen, Korea) and incubated for three to five days (P1 viral stock). Generated P1 viral stock was infected to Sf9 cells and incubated for two to four days (P2 viral stock). The same procedure was carried out to generate P3 viral stock. The thirdly propagated baculoviruses were infected into High Five cells (Invitrogen, USA) and incubated for five to seven days. Cell supernatant containing expressed recombinant proteins was collected, equilibrated, and filtered. The equilibrated culture supernatant was incubated with IgG Sepharose resin (GE Healthcare Life Science, USA) for 30–60 min at 4°C with agitation. The protein-bound resin was loaded into a column and washed several times with 10X volumes of cold equilibrium buffer (10 mM sodium phosphate, 150 mM NaCl, pH 8.0). The bound proteins were eluted with 100 mM Glycine and 500 mM NaCl, pH 2.7 and instantly neutralized with 2 M Tris-Cl buffer (pH 8.8). The eluted proteins were then dialysed in in cold PBS buffer, pH 8.5 at 4°C and concentrated with centrifugal filter device (Amicon, Millipore, USA). Quantified proteins were used for SDS-PAGE, Western blot analysis, and the measurement of enzymatic activity.

### Detection of endogenous and recombinant calpain proteins

To confirm the presence of endogenous and recombinant calpain proteins, Western blot analysis was performed. Electrophoresed polyacrylamide gel was transferred onto a nitrocellulose membrane (Hybond-ECL, Amersham Bioscience, USA). The membrane was blocked with 5% skim milk and incubated either with a polyclonal anti-His antibody (1:5,000 dilution) or an anti-*Pf*-calpain antibody (anti-*Pf*-CapnA; AbFrontier, Korea) (1:2,000 dilution) generated from previous study [27] for two hours at room temperature. The blot was further incubated with goat anti-mouse conjugated-HRP antibodies for one hour (1:5,000 dilution).

### Measurement of calpain activity using the fluorogenic substrate assay

This assay was performed with Succinyl-Leu-Leu-Val-Tyr-7-amino-4-methylcoumarin (Suc-LLVY-AMC) substrate according to Debiasi *et al*. [28]. Recombinant *Pf*-calpains were incubated with 20 μM Suc-LLVY-AMC in a total volume of 200 μl with 100 mM Tris–HCl pH 7.5, 1 mM DTT for one hour at 37°C. For the inhibition assay, 100 μM protease inhibitors were pre-incubated with r*Pf*-calpains for one hour at 37°C before adding substrate. The fluorescence of AMC (aminomethylcoumarin) was measured in one hour after the addition of the substrates using a fluorometer (Infinite F200, Tecan, Switzerland) with excitation at 360 nm and emission at 460 nm. The pH-optimum of r*Pf*-calpains was tested using this fluorogenic-substrate assay at various pH conditions ranging from pH 4.5 to pH 9.5: 100 mM sodium acetate buffer was used for pH 4.5 or pH 5.5 conditions, and 100 mM Tris–HCl buffer was used for pH 6.5, pH 7.5, pH 8.5 and pH 9.5.

### Measurement of calpain activity using gelatin zymography

SDS-PAGE was performed using 10% polyacrylamide gels containing 0.1% gelatin as a copolymerized substrate under non-reducing conditions. The protein samples were prepared by mixing with Tris-glycine-SDS sample buffer without 2-mercaptoethanol for 10 min at room temperature. Following protein separation, in order to remove SDS, the gel was incubated in 2.5% Triton X-100 solution with vigorous agitation for one hour at room temperature, and then incubated in zymogram developing buffer containing 50 mM Tris–HCl, 0.2 M NaCl, 5 mM CaCl_2_ and 2.5% Triton X-100 overnight at 37°C. For the inhibition assay, 100 μM protease inhibitors were diluted in zymogram developing buffer. Finally, the gel was stained with Coomassie blue R-250 and followed by destaining. The proteolytic activity of r*Pf*-calpains was determined by observing clear zones on SDS gels.

### Homology modeling of *Pf*-calpain subdomain IIa

The primary sequence of *Pf*-calpain (accession: HQ386136), which was determined in previous report [27], was used as a query to search the most appropriate template for homology modeling. Using the non-redundant (nr) protein sequence database taken from the National Center for Biotechnology Information [29], BLAST (basic local alignment search tool) [30] search was performed. Among the searched proteins whose X-ray crystal structures are available, human calpain 8 (*Hs*-calpain 8) showed the highest sequence identity with *Pf*-calpain, and its active form complexed with leupeptin and Ca^2+^ ions was selected as a template (2NQA.pdb).

Based on the primary sequences of *Pf*-calpain and the selected template *Hs*-calpain 8, multiple sequence alignment was performed with CLUSTAL W2 which uses a progressive pairwise alignment algorithm [31]. Since the highly conserved cysteine, histidine, and asparagine residues are reported to form the catalytic triad in the catalytic site of calpain [27], these catalytic triads of *Pf*-calpain and *Hs*-calpain 8 were focused on matching. Initially, sequences of *Pf*-calpain and *Hs*-calpain 8 were aligned, but a reasonable result with well-matched catalytic triads was not obtained. Therefore, sequence alignment in two steps was carried out using *Hs*-calpain 7 as a reference, because it is reported that the *Pf*-calpain genome exhibits high sequence similarity to *C. elegans* calpain 7 [22], and the BLAST search result showed that *Hs*-calpain 7 possessed high sequence identity to *Pf*-calpain. Firstly, two sets of sequences were aligned, one contains *Pf*-calpain, *Pv*-calpain (accession: HQ386136), and *Hs*-calpain 7 (accession: NP055111); and the other consists of *Hs*-calpain 7 and *Hs*-calpain 8 (PDB id: 2NQA). Then, the sequences of *Pf*-calpain and *Hs*-calpain 8 were manually aligned using the aligned sequence of *Hs*-calpain 7 as a reference.

Using the multiple sequence alignment, the homology model of *Pf*-calpain was built by MODELER v.9.4 program in Discovery Studio v.3.5 (Accelrys Software Inc., USA). The validity of the models and compatibility of each amino acid residue in the given 3D environment were checked by Verify Protein (Profiles-3D) protocol in Discovery Studio (Additional file 1 and 2). Among the resulting 50 models, the most appropriate model, i.e., one with the low probability density function (PDF) total energy, high verify score, and adequate orientation of the catalytic triad residues, was chosen for the loop refinement process. All hydrogen atoms were added to the refined model, and the protonation state of the ionizable groups was chosen appropriate to pH 7.4 including the protonated His1179 [32]. Then, the model was further refined by the 1000 ps molecular dynamics (MD) simulation and energy minimization with CHARMm force field. During the simulations, the implicit solvent model was applied using generalized born with molecular volume (GBMV) method [33]. The final model was evaluated by verify protein (Profiles-3D) and ERRAT score from the structure analysis and verification server (SAVES) [34].

### Docking of ALLN onto the active site of *Pf*-calpain subdomain IIa

The 3D structure of ALLN was generated with Concord and energy minimized using MMFF94s force field and MMFF94 charge until the rms of Powell gradient was 0.05 kcal/mol·Å in SYBYL v.8.1.1 (Tripos Int., USA). The docking study on the minimized homology model of *Pf*-calpain was carried out using GOLD v.5.1 (Cambridge Crystallographic Data Centre, UK), which employs a genetic algorithm (GA). The binding site was defined as 12 Å around the sulphur atom of Cys1035, and the side chains of Cys1035 and His1179 were set to be flexible with ‘crystal mode’.

For the covalent docking, the hydrogen atom attached to the sulphur atom of Cys1035 was removed, and the hydrogen atom in the aldehyde group of ALLN was replaced to sulfur. Then, the sulfur atoms of Cys1035 and ALLN were set as the protein link atom and ligand link atom, respectively. The carbonyl oxygen of ALLN and the delta-position hydrogen of His1179-imidazole were constrained to form a hydrogen bonding interaction. GoldScore scoring function was used and other parameters were set as defaults except that the number of GA runs was 50. The resulting docked complexes were energy minimized using the CHARMm force field and the implicit solvent model, GBMV, until the rms of conjugate gradient was lower than 0.0001 kcal/mol·Å.

### Molecular dynamics simulation of *Pf*-calpain subdomain IIa complexed with ALLN

Molecular dynamics (MD) simulation was performed by the CHARMm program, implemented in Discovery Studio v.3.5, with CHARMm force field and cff partial charge. The complex structure of *Pf*-calpain and covalently-bound ALLN was solvated by water molecules with orthothrombic cell shape of the explicit periodic boundary model. The solvated system was gently minimized with steepest descent algorithm until the tolerance reached 0.1 kcal/mol·Å, and further minimized by conjugate gradient algorithm until the tolerance reached 0.0001 kcal/ mol·Å. The minimized system was gradually heated to 300 K, and then, it was followed by the equilibration step for 300 ps. Finally, the production phase was carried out during 3000 ps using an NPT ensemble at 300 K. During the MD simulations, the integration time step of 1 fs was used, and the SHAKE constraints were applied.

All computation calculations were undertaken on an Intel® XeonTM Quad-core 2.5 GHz workstation with Linux Cent OS release 5.5. Sequence alignment, homology modeling, loop refinement, energy minimization and molecular dynamics were performed in Discovery Studio v.3.5 (Accelrys Inc., USA). Figures are generated by the SYBYL v.8.1.1 and PyMOL Molecular Graphics System v.1.3.

## Results

### The catalytic domain IIa is sufficient for enzymatic activity of *Pf*-calpain

**Figure 1:**
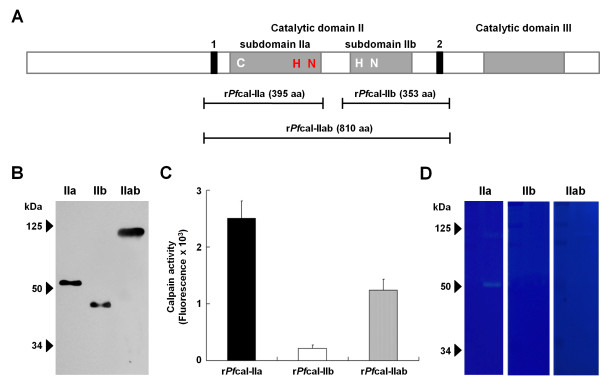
**The enzymatically active form of *Pf*-calpain is identified. A**) Schematic diagram of the full-length *Pf*-calpain and three recombinant *Pf*-calpain constructs. Cys 1035, His 1371, and Asn 1391 were indicated in catalytic subdomain IIa and IIb, respectively. The predicted histidine (His 1179) and asparagine (Asn 1195) residues were shown in red. These residues were further analysed. Black boxes indicate the recognition sites of anti-*Pf*calpain antibodies. Left, anti-*Pf*CapnA antibody; Right, anti-*Pf*CapnB antibody. **B**) Western blot of purified recombinant *Pf*-calpains using an anti-*Pf*-calpain antibodies. **C**) Calpain enzymatic activity measured by the fluorogenic substrate assay. **D**) Calpain enzymatic activity measured by gelatin zymography.

To characterize the *Plasmodium* calpain protein, the full length genomic *Pf*-calpain gene (6.2 kb) was firstly cloned from *P. falciparum* FCR-3 strain (Figure 1A; Accession No. HQ386136). Multiple alignments revealed that *Pf*-calpain protein of *P. falciparum* FCR-3 strain (Accession No. ADQ00190.1) is 98% identical to that of *P. falciparum* 3D7 strain (Accession No. ABR18792.1). As described elsewhere [22,23], *Pf*-calpain belongs to atypical calpain family, and has the highest similarity with mammalian calpain-7 (Accession No. NP_055111) and *C. elegans* calpain-7 (Accession No. NP_497964). They do not have regulatory subunits and thus they are considered to have Ca^2+^-independent calpain activity. However, crucial amino acid residues are well conserved in the catalytic subdomain IIa (cysteine) and IIb (histidine and asparagine).

To address the activation mode of *Pf*-calpain, three kinds of recombinant calpain genes were designed based on the full length genomic *Pf*-calpain gene (6.2 kb). Each recombinant *Pf*-calpain includes catalytic subdomain IIa (r*Pf*cal-IIa), catalytic subdomain IIb (r*Pf*cal-IIb), and catalytic subdomain IIab (r*Pf*cal-IIab), respectively. Recombinant proteins were successfully expressed and purified in bacterial system. The proteins were confirmed with western blot analysis using anti-*Pf*CapnA and anti-*Pf*CapnB antibodies, which can specifically recognize *Pf*-calpain. The predicted size of recombinant proteins was approximately 50 kDa for r*Pf*cal-IIa, 40 kDa for r*Pf*cal-IIb, and 90 kDa for r*Pf*cal-IIab (Figure 1B).

The activity of recombinant *Pf-*calpains was determined either with the fluorogenic substrate assay using Suc-LLVY-AMC substrate or with gelatin zymography. Interestingly and very surprisingly, recombinant protein having only catalytic subdomain IIa (r*Pf*cal-IIa) displayed the highest enzymatic activity compared to the others (Figure 1C and 1D). The fluorescence of substrates was apparently increased and the proteolytic activity against gelatin was obviously seen on SDS gel. However, r*Pf*cal-IIb did not exhibit any enzymatic activity in both assays and r*Pf*cal-IIab showed relatively lower enzymatic activity. Thus, r*Pf*cal-IIa protein was further analysed.

**Figure 2:**
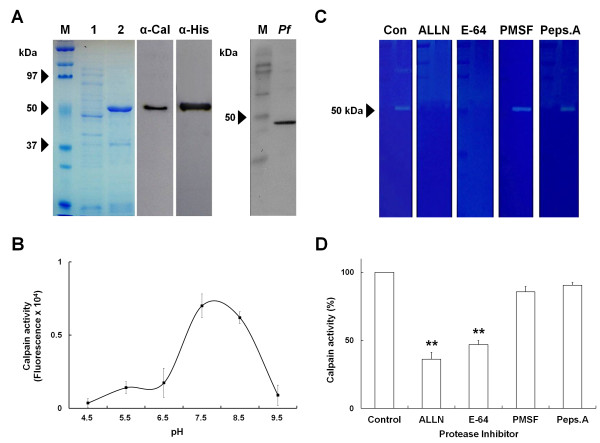
**The catalytic subdomain IIa is sufficient to have enzymatic activity of *Pf*-calpain. A**) Purified recombinant *Pf*cal-IIa (r*Pf*cal-IIa) and endogenous *Pf*-calpain were confirmed by Western blot analysis using anti-*Pf*CapnA antibody (α-Cal) and anti-poly-His Tag antibody (α-His). M, size marker; lane 1, un-induced *E. coli* cell lysate; lane 2, purified r*Pf*cal-IIa; *Pf*, protein extracts of *P. falciparum*. **B**) Effect of pH on the activity of r*Pf*cal-IIa was assessed by fluorogenic substrate assay. The activity of r*Pf*cal-IIa and the effects of protease inhibitors were analysed by **C**) gelatin zymography and **D**) the fluorogenic substrate assay. **C**) The proteolytic activity is shown in clear zone on SDS gels. **D**) Data are presented as mean ± S.D. Statistical analysis was performed using the student’s *t*-test. **p < 0.01.

Very interestingly, the protein size of r*Pf*cal-IIa was nearly identical to that of endogenous calpain fragment, which was detected from *P. falciparum* whole protein extracts (Figure 2A). Although the entire *Pf*-calpain protein is supposed to be expressed with approximately 225 kDa, the 46 kDa calpain fragment was only detected from whole lysate of parasites with anti-*Pf*-calpain antibody. This indirectly suggests that the form of *Pf*cal-IIa could be actually active *Pf*-calpain *in vivo*. However, it cannot rule out the possibility of autolysis and/or degradation of calpain protein during preparation.

The optimal pH condition for r*Pf*cal-IIa activity was further tested. The pH condition ranging from 4.5 to 9.5 was used to elucidate the optimal pH of purified r*Pf*cal-IIa protein (Figure 2B). As shown in Figure 2B, the fluorogenic substrate assay revealed that r*Pf*cal-IIa protein is the most active at pH 7.5. Thus, pH 7.5 was used as the optimal pH condition for the subsequent assays. Next, the enzymatic activity of r*Pf*cal-IIa protein was checked with the addition of various protease inhibitors; ALLN as a calpain inhibitor, E-64 as a common cysteine protease inhibitor, PMSF as a serine protease inhibitor, and Pepstatin A as an aspartic protease inhibitor. Both fluorogenic substrate assay and gelatin zymography showed that the enzymatic activity of r*Pf*cal-IIa protein was significantly inhibited by ALLN and E-64 (Figure 2C and 2D). These results confirmed that r*Pf*cal-IIa protein is indeed the active form of *Pf*-calpain and thereby its activity is inhibited by a broad cysteine protease inhibitor, E-64. More interestingly, a calpain inhibitor, ALLN could effectively inhibit *Pf*-calpain, supporting the possibility that *Pf*-calpain may contribute to the inhibition of malaria parasite proliferation under ALLN treatment.

### *Pf*-calpain may act in monomeric form

It has been well documented that the catalytic subdomain IIa and IIb play a key role for calpain enzymatic activity because of catalytic triad formed among amino acid residues in those subdomains [22,35]. In the presence of Ca^2+^, two catalytic subdomains become structurally closer and thereby cysteine residue in the subdomain IIa and histidine-asparagine residues in the subdomain IIb form a functional catalytic site [35]. However, *Pf*-calpain is considered to be Ca^2+^-independent. Furthermore, in this study, it is shown that the catalytic subdomain IIa is sufficient to have an enzymatic activity of calpain. Thus, the next question addressed is how *Pf*-calpain becomes active.

**Figure 3:**
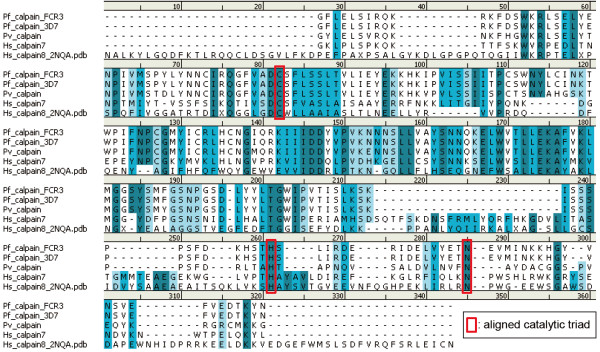
**Multiple sequence alignment of the catalytic subdomain IIa.***Plasmodium falciparum* (FCR-3) calpain (GenBank, ADQ00190.1), *P. falciparum* (3D7) calpain (GenBank, ABR187921.1), *Plasmodium vivax* calpain (GenBank, EDL46679.1), and *Homo sapiens* calpain-7 (GenBank, NP_055111) were aligned using Crystal W2 program. *P. falciparum* (FCR-3) calpain was 33.6% identical to *H. sapiens* calpain-7 and 73.5% identical to *P. vivax* calpain. The predicted histidine (His 1179) and asparagine (Asn 1195) residues were shown in red with cysteine (Cys 1035).

First of all, the amino acid sequence of catalytic subdomain IIa was analysed. Multiple sequence alignment was performed using the sequences of *Pf*-calpain, *Pv*-calpain and *Homo sapiens* calpain-7 (Figure 3). Interestingly, it was predicted that the catalytic subdomain IIa contains plausible histidine (His1179) and asparagine (Asn1195) residues necessary for forming catalytic triad. This may explain how *Pf*-calpain is active without Ca^2+^ stimulation. To test whether those histidine and asparagine residues are functional or not, two different approaches were conducted. One is molecular modeling and the other is molecular technique introducing mutations on those amino acid residues.

### Homology model of *Pf*-calpain subdomain IIa appears to well construct the active site residues for catalysis

In order to predict the structure of *Pf*-calpain, homology modelling study was conducted. The X-ray crystal structure of *Hs*-calpain 8 (PDB id: 2NQA) was selected as a template, since it shows the minimum E-value at the BLAST search of *Pf*-calpain sequence in the protein data bank. The sequence identity and similarity between *Pf*-calpain and *Hs*-calpain 8 are 16% and 31%, respectively. Based on the multiple sequence alignment (Figure 3), homology modelling was done by MODELER, and the most reasonable model was refined by Loop Refinement protocol in MODELER. The resulting model was further refined by the molecular dynamics (MD) simulation and energy minimization.

**Figure 4:**
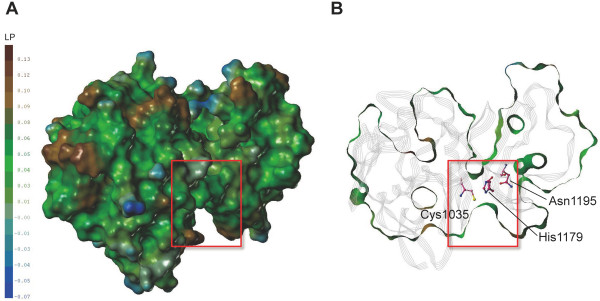
**Homology model of *Pf*-calpain subdomain IIa. A**) Molecular surface of the model. Connolly surface was generated by MOLCAD and colored by lipophilic potential (LP). **B**) The z-clipped surface of the model and its secondary structure with gray line ribbon representation. The catalytic triad residues are represented as sticks, and the catalytic cleft is marked with the red box. Figure is generated by the SYBYL v.8.1.1.

**Figure 5:**
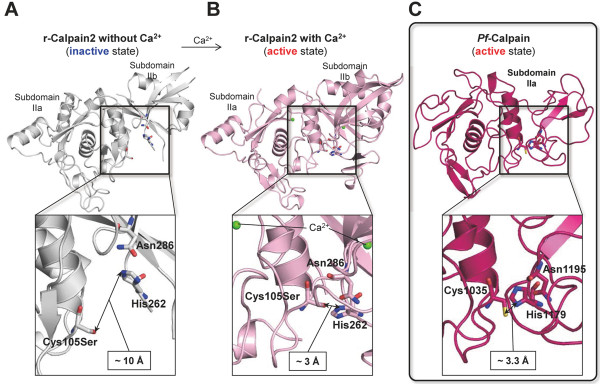
**Comparison of the *Pf*-calpain model to the active and inactive forms of the typical calpain. A**) Rat calpain 2 without Ca^2+^ ion (1U5I.pdb). **B**) Rat calpain 2 complexed with Ca^2+^ ions (1MDW.pdb). **C**) The homology model of *Pf*-calpain subdomain IIa. Catalytic triad residues are displayed in sticks and their residue numbers are marked. Figure is generated by the SYBYL v.8.1.1.

The overall topology of the *Pf*-calpain subdomain IIa homology model shows a globular shape and the active site is well constructed for the enzyme function (Figure 4). In case of the typical calpains, the activation state is dependent on the Ca^2+^ binding [36]. In the absence of Ca^2+^, the typical calpains are inactive with their subdomains IIa and IIb distant to each other (Figure 5A). Upon the Ca^2+^ binding, the subdomains IIa and IIb become quite closer and make the catalytic triad properly oriented for catalysis (Figure 5B). Interestingly, the homology model of *Pf*-calpain subdomain IIa consists of two lobes, and it forms the active site containing the plausible catalytic triad residues, Cys1035, His1179, and Asn1195, close to one another (Figure 5C).

The first lobe (residues from Gly990 to Ile1156) of the model is very similar to the catalytic subdomain IIa of the typical calpain structures. The catalytic Cys1035 is located in this lobe, and its side chain faces toward the cleft region between the two lobes. In addition, Gln1029 interacts with the backbone NH of Cys1035, forming the oxyanion hole, which is reported to stabilize the catalytic intermediate [32]. The second lobe (residues from Pro1157 to Asn1218) contains His1179 and Asn1195, which are properly positioned to make the hydrogen bond network with Cys1035, reflecting their roles as the catalytic triad. Unlike the typical calpains, *Pf*-calpain is sufficiently enough to function with the subdomain IIa only, and this result could explain why it has catalytic activity independent to Ca^2+^ binding.

**Figure 6:**
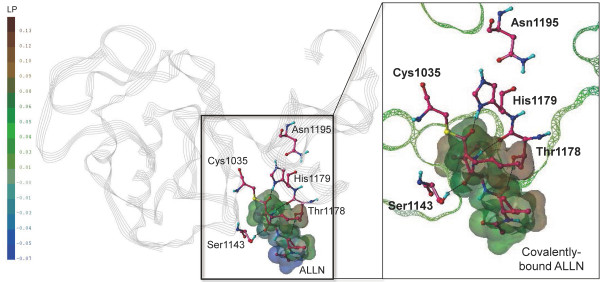
**Refined model of *Pf*-calpain subdomain IIa covalently bound with ALLN.** The secondary structure of *Pf*-calpain is displayed in grey line ribbon. The catalytic triad residues, Cys1035, His1179, and Asn1195, are displayed in ball-and-stick with the carbon color of hotpink. ALLN, covalently bound with Cys1035, was represented in ball-and-stick with the carbon color of hotpink, and its van der Waals surface was created by MOLCAD. In the enlarged view, the Connolly surface of *Pf*-calpain is displayed in mesh, and z-clipped from back and forth for the visual convenience. The molecular surfaces of the protein and ligand are colored by lipophilic potential (LP). Figure is generated by the PyMOL Molecular Graphics System v.1.3.

ALLN showed the inhibitory activity onto *Pf*-calpain [14,15,26]. To predict its binding mode, the covalent docking of ALLN was performed into the active site of *Pf*-calpain model using GOLD program. The complex structure was further refined by molecular dynamics (MD) simulation in the explicitly solvated system. ALLN was nicely occupied the cleft between the two lobes of *Pf*-calpain and made the covalent bond with catalytic Cys1035 (Figure 6). It also formed the H-bonding interactions with Ser1143, Thr1178, and His1179, explaining its good inhibitory activity onto *Pf*-calpain.

### Point mutation study confirmed that the catalytic triad may be formed in the catalytic subdomain IIa

**Figure 7:**
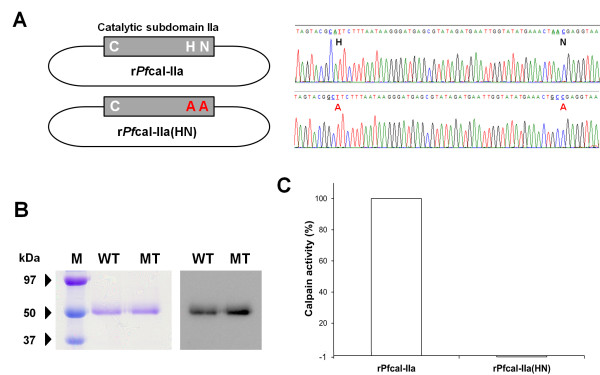
**The predicted histidine and asparagine residues are functional. A**) Histidine and asparagine residues were alanine-substituted and confirmed by sequencing. Targeted amino acids were underlined in chromatogram. **B**) Both wild-type form of r*Pf*cal-IIa (WT) and mutant form of r*Pf*cal-IIa (MT) were expressed in the baculovirus system. Purified proteins were confirmed by Western blot using anti-*Pf*CapnA antibody. **C**) The enzymatic activity was measured by fluorogenic substrate assay. The mutations in predicted histidine and asparagine residues completely disrupted enzymatic activity of r*Pf*cal-IIa.

Plausible histidine and asparagine residues in the catalytic subdomain IIa were point mutated to see their functionality. Two amino acid residues were substituted by alanine (Figure 7A). The recombinant *Pf*cal-IIa gene (r*Pf*cal-IIa) and the mutated recombinant *Pf*cal-IIa gene (r*Pf*cal-IIa(HN)) were cloned and successfully expressed in insect cell system (Figure 7B). Purified recombinant proteins were tested for their enzymatic activity using the fluorogenic substrate assay. Intriguingly, the mutated r*Pf*cal-IIa proteins (r*Pf*cal-IIa(HN)) did not show any enzymatic activity compared to normal form (r*Pf*cal-IIa) (Figure 7C). The enzymatic activity was dramatically reduced with the mutations in two amino acid residues. These suggest that two amino acid residues in the catalytic subdomain IIa are actually functional. This supports the possibility that *Pf*-calpain may act in monomeric form.

## Discussion

With the increasing resistance of malaria parasites to available drugs [3,4], there is a need to develop new anti-malarial drugs. Although *Plasmodium* plasmepsin and falcipain have been targeted for developing anti-malarial drugs [5,16-19], it has been revealed that a common aspartic proteases inhibitor and cysteine proteases inhibitor cannot completely inhibit plasmepsin and falcipain activity [5,20]. Meanwhile, ALLN, a calpain inhibitor has been proposed to inhibit plasmepsin and falcipain [14,15]. Nonetheless, it is still viable to reason the probability that calpain could be one of the mediators for haemoglobin degradation [26]. *Pf*-calpain was identified from *P. falciparum* FCR-3 strain with high identity to a putative *Pf*-calpain previously reported (3D7 strain, MAL13P1.310) [24,27]. The catalytic domain II of newly isolated *Pf*-calpain has high sequence similarity to the calpain-7 family that contains three active sites (Cys1035, His1371 and Asn1391). These three sites constitute a cleft crucial for a catalytic activity [22,23]. A Cys-His-Asn triad, with a high degree of identity to human calpains is observed in its vicinity, reflecting stringent functional and mechanistic conservation [22].

In this study, for the first time, the active form of *Pf*-calpain was identified. Surprisingly, the active form of *Pf*-calpain was revealed to consist only of the catalytic subdomain IIa. This result was unexpected because the catalytic subdomain IIa is supposed to have an incomplete Cys-His-Asn triad structure. However, all evidences resolved in this study propose that *Pf*-calpain is sufficiently active only with the catalytic subdomain IIa; 1) endogenous *Pf*-calpain was detected from *P. falciparum* whole extracts with the similar size to r*Pf*cal-IIa; 2) r*Pf*cal-IIa showed the strongest enzymatic activity and this activity was effectively inhibited by E-64 and ALLN; 3) the catalytic subdomain IIa is predicted to have plausible histidine and asparagine residues; 4) the mutations of those amino acid residues completely disrupted *Pf*-calpain activity; and 5) finally, molecular modeling study predicted the molecular structure of *Pf*-calpain subdomain IIa, and it supports that Cys1035, His1179, and Asn1195 residues in subdomain IIa are positioned quite close to one another, forming the catalytic triad with the appropriate orientation for catalysis.

*Pf*-calpain seems to be activated by different way from mammalian typical calpains. Since *Pf*-calpain does not have regulatory subunits (domain V and VI), it may function as a monomeric form. In addition, since *Pf*-calpain does not have domains responsible for Ca^2+^ binding (domain IV in the catalytic subunit and domain VI in the regulatory subunit), Ca^2+^ may not contribute to the conformational change leading to the closer positioning of catalytic subdomain IIa and IIb, which have important amino acid residues necessary for catalytic triad formation. These results propose the possible activation mode of *Pf*-calpain. The catalytic subdomain IIa has all three functional amino acid residues, and the homology model of *Pf*-calpain showed that the catalytic site can be formed with subdomain IIa only, reflecting the catalytic activity independent to Ca^2+^ binding. Although *Hs*-calpain 8 was utilized as a template to analyse homology model, *Pf*-calpain possesses only 16% identity with *Hs*-calpain 8, providing *Pf*-calpain as a parasite-specific target. Furthermore, the distinct monomeric structure of calpain is highly specific for malaria parasite. Thus, *Pf*-calpain could be served as a novel target to develop parasite specific anti-malarial drugs. In that point of view, the identification of enzymatically active *Pf*-calpain might be the starting point to establish high-throughput screening system for *Pf*-calpain-based drug development. In addition, the monomeric structure of *Pf*-calpain may provide tremendous advantages to synthesize candidate compounds by simplifying the synthesis steps.

Recent reports revealed that calpain may localize in nucleolus and/or cytoplasm [27,37]. The transcription level of calpain is highly expressed in schizont stage [22], but anti-*Pf*-calpain antibodies detect calpain expression throughout the developmental stages from early ring to late schizont [27]. It has been involved in cell cycle progression during trophozoite development [24]. However, the biological functions of *Pf*-calpain are still largely unknown. In this study, biochemical and structural properties of *Pf*-calpain was uncovered. The neutral *Pf*-calpain provokes a critical question. How this neutral enzyme could be involved in haemoglobin hydrolysis, if is the case? It is well known that haemoglobin degradation occurs in acidic food vacuole primarily by acidic proteases [5,6]. Thus, it is unclear yet whether *Pf*-calpain mediates haemoglobin degradation or not. If it is involved in haemoglobin metabolism, then it is also inconclusive whether it is in a way through plasmepsin and falcipain or in an independent way. Further functional studies will be needed.

## Competing interests

The authors declare that they have no competing interests.

## Authors’ contributions

HP and SC conceived and designed the experiments. BS, HS, YL, JL, KK, BL, YC and JC performed the experiments. BS, HS, YL and JL analysed the data. HP, SC and JC contributed reagents/materials/analysis tools. BS, HS, YL, JL, HP and SC wrote the manuscript. The authors have read and approved the final manuscript.

## Supplementary Material

Additional file 1 **Quality assessment of the model. A**) Verify scores and **B**) ERRAT scores of the template X-ray crystal structure (left) and the refined homology model (right). Click here for file

Additional file 2 Quality assessment of the homology model. Click here for file
