# Supplementary figures and images for: Identification of active Plasmodium falciparum calpain to establish screening system for Pf-calpain-based drug development
Source: Malar J. 2013 Feb 4;12:47. doi: 10.1186/1475-2875-12-47 (PMC3583800; doi:10.1186/1475-2875-12-47)

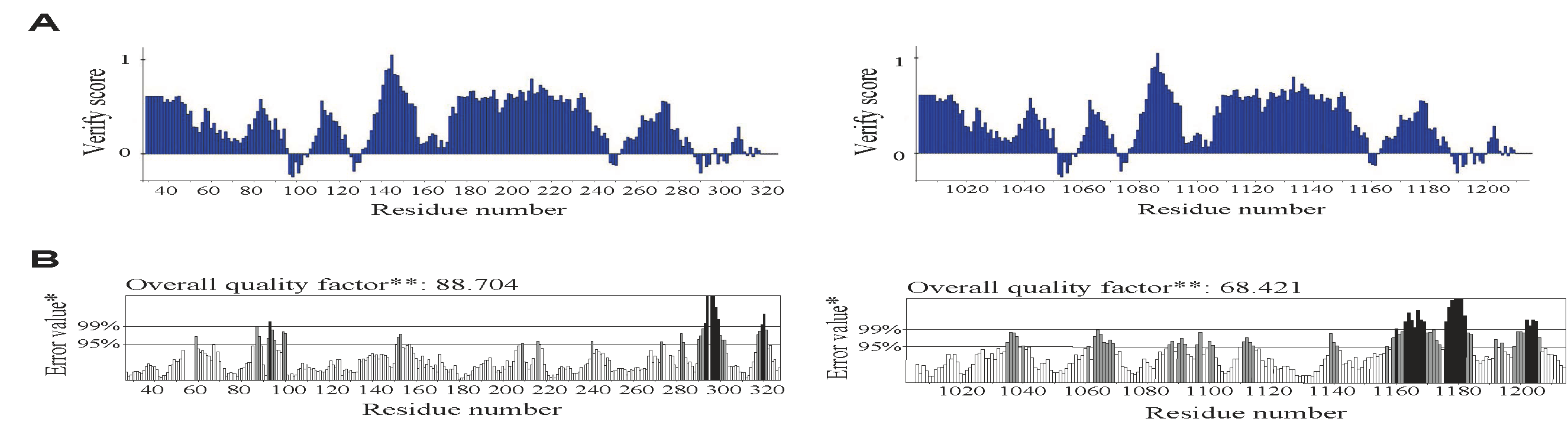

Supplement: Additional file 1 — Quality assessment of the model. A) Verify scores and B) ERRAT scores of the template X-ray crystal structure (left) and the refined homology model (right). [file 1475-2875-12-47-S1.tiff]
