# Supplementary material for: Identification of active Plasmodium falciparum calpain to establish screening system for Pf-calpain-based drug development
Source: Malar J. 2013 Feb 4;12:47. doi: 10.1186/1475-2875-12-47 (PMC3583800; doi:10.1186/1475-2875-12-47)
Supplement: Additional file 2 — Quality assessment of the homology model. [file 1475-2875-12-47-S2.docx]

**Additional file 2**

**Quality assessment of the homology model**

The quality of the final homology model was assessed by Verify Protein (Profiles-3D) protocol in Discovery Studio v.3.5 (Accelrys Software Inc., San Diego, CA, USA). The verify score of our model is 63.62, which is much higher than the verify expected low score (i.e., 44.3197) for that size of proteins (Additional file 1A). Also, the model was checked by ERRAT score from the Structure Analysis and Verification Server (SAVES) [1]. The ERRAT score could give an overall quality factor for non-bonded atomic interactions, and a score of greater than 50 is acceptable [1,2]. The template and our final refined model yielded ERRAT scores of 88.704 and 68.421, respectively, and the values were clearly well within the range of high quality (Additional file 1B). Overall, the verify score and ERRAT analysis indicated that the backbone conformation and non-bonded atomic interactions of our refined homology model for *Pf*-calpain subdomain IIa are all well within the acceptable range.

**References**

1. **The Structure Analysis and Verification Server**: http://nihserver.mbi.ucla.edu/SAVES/.

2. Colovos C, Yeates TO: **Verification of protein structures: patterns of nonbonded atomic interactions.** *Protein Sci* 1993, **2:**1511-1519.
